# Study of the Influence of PCL on the In Vitro Degradation of Extruded PLA Monofilaments and Melt-Spun Filaments

**DOI:** 10.3390/polym13020171

**Published:** 2021-01-06

**Authors:** Vivien Barral, Sophie Dropsit, Aurélie Cayla, Christine Campagne, Éric Devaux

**Affiliations:** 1 ENSAIT, GEMTEX—Laboratoire de Génie et Matériaux Textiles, F-59000 Lille, France, 2 Allée Louise et Victor Champier, 59056 Roubaix CEDEX 1, France; aurelie.cayla@ensait.fr (A.C.); christine.campagne@ensait.fr (C.C.); eric.devaux@ensait.fr (É.D.); 2 MATERIA NOVA—R&D CENTER, Avenue Nicolas Copernic 3, 7000 Mons, Belgique; sophie.dropsit@materianova.be

**Keywords:** biodegradable polymers, polyesters, immiscible blend, extrusion, melt spinning

## Abstract

This work presents the effect of a melt-spinning process on the degradation behavior of bioresorbable and immiscible poly(d,l-lactide) (PLA) and polycaprolactone (PCL) polymer blends. A large range of these blends, from PLA_90_PCL_10_ (90 wt% PLA and 10 wt% PCL) to PLA_60_PCL_40_ in increments of 10%, was processed via extrusion (diameter monofilament: ∅ ≈ 1 mm) and melt spinning (80 filaments: 50 to 70 µm each) to evaluate the impact of the PCL ratio and then melt spinning on the hydrolytic degradation of PLA, which allowed for highlighting the potential of a textile-based scaffold in bioresorbable implants. The morphologies of the structures were investigated via extracting PCL with acetic acid and scanning electron microscopy observations. Then, they were immersed in a Dulbecco’s Modified Eagle Medium (DMEM) media at 50 °C for 35 days and their properties were tested in order to evaluate the relation between the morphology and the evolution of the crystallinity degree and the mechanical and physical properties. As expected, the incorporation of PCL into the PLA matrix slowed down the hydrolytic degradation. It was shown that the degradation became heterogeneous with a small ratio of PCL. Finally, melt spinning had an impact on the morphology, and consequently, on the other properties over time.

## 1. Introduction

Nowadays, there is an emergent interest in degradable and bioresorbable polymers for their applications in the medical field, and especially for tissue engineering. They are classified according to geometrical criteria: one-dimensional structures, such as surgical sutures and ligatures [1]; two-dimensional structures, such as hernia repair meshes and sewing rings for heart valves prosthesis [1]; three-dimensional structures, such as walled tubular constructs for vascular grafts and orthopedic implants [1]. A very famous application example is the development of absorbable stents [2,3]. For tissue regeneration, the chosen material has to satisfy many criteria, such as biocompatibility or full resorbability i.e., their degradation products have to be metabolized and excreted. Most of the used polymers for these kinds of applications are an aliphatic polyester, such as polycaprolactone (PCL) [4,5,6], polybutylene succinate (PBS) [7], polylactide (PLA) [2,5], polyglycolide (PGA) [5], and poly-3-hydroxybutyrate (PHB, P3HB) [8]. Some others display ether–ester functions, such as polydioxanone (PDS, PDO) or polyethylene glycol (PEG). All these polymers have good biocompatibility and are known to be fully bioresorbable with different kinetic degradation times in the body.

The design of a 3D scaffold requires the structure itself to have an architecture that promotes the formation of native anisotropic tissue [9]; in other words, the structure has to act as a template for tissue growth by dictating the shape [1]. The structure must be a network of large interconnected pores or “macropores” with a minimum size of 100 μm. This network is necessary to allow for cell migration through the scaffold and promote nutrient and waste exchange [9,10]. As a consequence, the material must have a suitable surface for cell attachment, proliferation, and extracellular matrix production, and appropriate mechanical properties to match those at the site of implantation [9]. When all these requirements are met, polymers become a good choice of material because they are easy to process. They can be used in a 3D scaffold with a porous filament network that is adapted to a tissue engineering application. Additionally, polymers can have high stiffness and toughness and interesting properties for implants exposed to compressive forces [10]. These performances synergize with the advantages of textile structures, such as being lightweight to limit the degradation products of the scaffold; their fibrous aspect being able to better match the biological tissues, which are fibrous themselves [1]; the diversity of structures, such as knitted fabric and woven and nonwoven fabric.

In this study, two immiscible polymers were chosen: PLA and PCL. The blend of both polymers has been intensively studied because their individual mechanical properties are very different: PCL exhibits a very high strain at break that can be explained by its very low glass transition temperature (−60 °C) and low Young’s modulus (under 1 GPa) [4,5], whereas PLA has a higher stiffness but is often described as brittle [2]. These two polymers also have very different degradation kinetics; this allows for tailoring the material properties over time, depending on the application. PLA and PCL have different degradation modes, as explained by Navarro-Baena et al. [11]; PLA degrades via chain scission without any mass loss until the fragmentation step, while PCL degrades via scission of the end groups of polymer chains, which leads to a slight mass loss. PCL is also known to improve the mechanical properties of neat PLA [12]; moreover, it was shown by Matta et al. [13] that the addition of PCL accelerates the crystallization rate of PLA. Some studies were carried out in order to understand how this blend degrades. Vieira et al. [12] worked on commercial PLA_90_/PCL_10_ filaments for surgical sutures and showed that the decrease in tensile strength follows the same trend as molecular weight. They also found that mass loss occurred very late in the degradation process. Fukushima et al. [14] showed the difference in degradation between biotic and abiotic conditions, highlighting the importance of microorganisms, especially for PCL degradation.

Grizzi et al. [15] studied the influence of the size of the material by comparing the molecular weight and mass decrease of plates, films, and microspheres, and highlighted that smaller devices degrade slower. The authors explained that this phenomenon is due to the carboxylic acid oligomers entrapped inside the matrix of big devices, contributing to the autocatalytic effect. This phenomenon depends highly on the degradable polymer used, which has pushed scientists to study the critical size that separates bulk degradation and surface degradation; this was done by Von Burkersroda et al., who gave a critical thickness for each polymer used in degradable devices [16], for example, 7.4 cm for polylactide and 1.3 cm for polycaprolactone. These studies on the impact of the dimensions of the device have to be coupled with work on the process, as it is known that the process parameters have an impact on the morphology of an immiscible blend; for instance, Padilla-Lopez et al. have shown the effect of a drawing and cooling method on the deformation of the dispersed phase [17]. Even if some studies were performed in vivo [4,5,7], the large majority of these polymer studies were carried out in vitro, mostly on PLA, PCL, PGA, PDO, and their blends [11,12,14,18,19]. One of the biggest challenges with these materials is controlling the mechanical behavior over time in accordance with the intended application.

The aim of this work was to achieve melt spinning of the PLA/PCL blend in a large range of compositions, and then compare every multifilament’s degradation to extruded monofilaments in order to point out differences in the mechanical degradation behavior. Incorporating PCL is a way to increase the PLA crystallinity degree, but so is melt spinning. Both methods are used in order to decrease the degradation of the blend. First, the morphological, thermal, and mechanical properties of each extruded monofilament were studied in order to highlight the impact of PCL incorporation on the PLA properties. The same study was performed on every melt-spun filament to emphasize the impact of melt spinning on the mechanical and thermal properties. Then, the aging of each polymer blend was performed by controlling their mechanical properties, crystallinity, and molecular weight. Therefore, the role of the morphology on the degradation behavior of the blend could be highlighted. The spinning step allowed us to study the spinnability of a wide range of PLA/PCL blends and the consequence of the spinning process on the yarns’ morphologies. Finally, the dimension parameter was considered based on the literature.

## 2. Materials and Methods

### 2.1. Materials

- The PLA INGEO 6202D was supplied by NatureWorks (Minnetonka, MN, USA). The characteristics of the PLA are M_n(PLA)_ = 112,890 g/mol, polydispersity index (Mw/Mn) = 2.07, and D isomer content: <2%. The glass transition temperature T_g(PLA)_ is between 55 and 60 °C.
- The PCL CAPA 6400 was supplied by Perstorp (Malmö, Sweden). The characteristics of the PCL are M_n(PCL)_ = 53,122 g/mol; polydispersity index (Mw/Mn) = 1.75, and glass transition temperature T_g(PCL)_ = −60 °C.
- The Gibco Dulbecco’s Modified Eagle Medium (DMEM) was supplied in solid form by Fischer Scientific (Waltham, MA, USA), where 1.5 g/L NaHCO_3_ was added in order to reach a neutral pH of 7.4 ± 0.2.
- The acetic acid was supplied by Fischer Scientific.

### 2.2. Processing

Different compounds were dried at 50 °C for 24 h and prepared using a co-rotating intermeshing twin-screw extruder from Thermo-Haake, supplied by Thermo Fisher Scientific (Waltham, MA, USA), PTW 16/25p (L/D = 25), with a rotation speed of 100 rpm. An optimized temperature profile was used between 60 and 180 °C. The obtained monofilament was directly cooled using an airstream on a conveyor belt and cut into pellets using a granulator. Some of the monofilament, from PLA_90_/PCL_10_ to PLA_60_/PCL_40_, as well as neat PLA and PCL, were saved for each material produced in order to perform degradation and mechanical tests.

A Busschaert engineering Spinboy I device, supplied by Busschaert Engineering (Nijverheidslaan, Belgium), was used for the melt-spinning process. The PLA/PCL blend pellets were dried at 50 °C for 24 h and processed through a single-screw extruder that was heated with a temperature profile between 159 and 175 °C. The compound went through a volumetric pump (52.5 cm^3^/min) and then through two spinnerets with 40 holes that were 1.2 mm in cross-section each in order to produce a continuous multifilament yarn. The material was then cooled down using an airstream and finally covered with spin finish oil. The multifilament was then drawn between a supply and a drawing roll (with speed and temperature combinations of V_1_, T_1_ and V_2_, T_2_) where V_1_ = 80 m/min, T_1_ = 25 °C and V_2_ = 120 m/min, T_2_ = 30 °C. Finally, the multifilament was wound on a roll. Several multifilaments (80 filaments) were produced with various compositions from PLA_100_ to PLA_60_/PCL_40_ per 10 wt% PLA_100_, PLA_90_/PCL_10_, PLA_80_/PCL_20_, PLA_70_/PCL_30_, and PLA_60_/PCL_40_.

### 2.3. Material Degradation Method

The in vitro degradation tests were carried out at 50 °C and 80% relative humidity in a climatic chamber for 35 days. Fifty centimeters of each multifilament was cut and immersed in DMEM media. With the same method, extruded monofilaments of 7 cm each were taken directly from the extrusion and were immersed in DMEM. After 7, 14, and 35 days, each extruded monofilament and multifilament was taken out to be characterized to observe the degradation via gel permeation chromatography (GPC), scanning electron microscopy (SEM), differential scanning calorimetry (DSC) analysis, and mechanical characterizations.

### 2.4. Characterization Methods

#### 2.4.1. Thermal Properties

DSC characterizations of the extruded monofilaments and melt-spun multifilaments were performed with 5 to 10 mg of dry material on a 2920 Modulated DSC from TA Instruments (New Castle, DE, USA) under a nitrogen flow of 50 mL/min. For each material, the procedure was as follows: a first heating scan at 10 °C/min from −20 °C up to 200 °C, an isotherm at this temperature for 3 min, then a scan at 10 °C/min down to −20 °C, an isotherm at this temperature for 3 min, and finally, a second heating scan from −20 °C to 200 °C at 10 °C/min. The first cycle was the most important because it contained the thermal history of the material and changed over time with degradation. The crystallinity degree (χc (%)) of PLA in each material was calculated according to the following Equation (1):

(1) $$\chi c\mathrm{PLA}\;\left(\%\right)=\frac{\left|\Delta \mathrm{Hm}\right|-\left|\Delta \mathrm{Hcc}\right|}{\mathrm{wPLA}\times \left|\Delta {\mathrm{Hm}}^{0}\right|}\times 100,$$

where wPLA is the PLA mass fraction in each blend; ΔHm^0^ is the reference enthalpy of PLA, which is defined as the melting heat flow of a 100% crystalline sample (93 J/g) [20,21]; ΔHm (J/g) is the melting enthalpy; ΔHcc (J/g) is the cold crystallization enthalpy.

#### 2.4.2. Morphological Properties

##### PCL Phase Extraction

The morphologies of the immiscible polymer blends were estimated using a selective phase extraction protocol of PCL. The polymer blend compounds and multifilaments were immersed in acetic acid for 4 h. Then, they were filtered and dried at 50 °C. The weight was measured after 24 h in a metrology laboratory at 21 °C and 65% relative humidity, and the immersion process was repeated until the mass tended toward a constant value. The PCL continuity or accessibility in the compound or the multifilament is linked to the total mass fraction before and after the protocol, as shown in the following equation:

(2) $$\mathrm{PCL}\;\mathrm{accessibility}\;\left(\%\right)=\frac{m_{i}-m_{f}}{\mathrm{wPCL}\times m_{i}}\times 100,$$

where *m_i_* is the initial mass (g), *m_f_* is the final mass (g), and wPCL is the polycaprolactone weight ratio.

##### Scanning Electron Microscopy

The morphologies of different blends, in both the cross-sectional and longitudinal views, were performed using a scanning electronic microscope Hitachi SU8020 SEM supplied by Hitachi high-technologies corporation (Tokyo, Japan). In order to facilitate the observations, a 5 nm gold layer was applied under vacuum on samples using a LEICA EM SCD050 (Wetzlar, Germany).

#### 2.4.3. Linear Density

The linear density (dTex: g/10 km) of each filament extracted from the multifilament was measured using a Zweigle vibroskop from Uster Technologies (NF G 07-306 norm, Uster, Switzerland) in order to observe the swelling behavior of the different materials. Supposing that every fiber cross-section is round, the linear density can be computed using the following equation:

(3) $$D=\sqrt{\frac{4\times 10^{10}\times T}{1.25\times \pi }},$$

where *D* represents the diameter of a single filament in μm, T the fineness in dTex, and 1.25 is the density of PLA. The Zwick 1456 device, supplied by Zwick Roell Group (Ulm, Germany), is able to convert linear density into a diameter, which is useful for converting every result into maximum stress (MPa) terms.

#### 2.4.4. Molecular Weight and Polydispersity Index

Size-exclusion chromatography (SEC) measurements were performed in chloroform (CHCl_3_) at 23 °C using a Polymer Laboratories Liquid Chromatograph equipped with a PL-DG802 degasser (Santa Clara, CA, USA), an isocratic high-performance liquid chromatography (HPLC) LC 1120 pump (flow rate = 1 mL min^−1^), a Marathon Autosampler (loop volume = 200 µL, solution concentration = 1 mg mL^−1^), a PL-DRI refractive index detector and three columns: a PL gel 10 µm guard column and two PL gel Mixed-B 10 µm columns (linear columns for the separation of molecular weights (MW) ranging from 106 to 500 daltons). Poly(styrene) (PS) standards were used for calibration.

#### 2.4.5. Mechanical Properties

##### Mechanical Properties of the Spun Melt

Mechanical tests were performed on an isolated monofilament that was extracted from a multifilament with a Zwick 1456 test device. According to the NF EN ISO 5079 standards (written in 1996, updated in 2020 [X]), the distance separating the clamps was 20 mm and the crossbar speed was 20 mm/min. The cell force on the bench was 10 N. Each material was tested 10 times and every test was performed in standard atmospheric conditions: a temperature of 20 ± 2 °C and a room humidity of 65 ± 5%. The main characteristics that were measured were the maximum stress (MPa), elongation at break (%), and Young’s modulus (GPa).

##### Mechanical Properties of the Extruded Monofilament

Mechanical tests on the extruded monofilament were performed with an MTS Criterion test device, supplied by MTS Systems Corporation (Eden Prairie, MN, USA), which was equipped with a 10 kN sensor. The distance separating the clamps was 50 mm and the crossbar speed was 50 mm/min. Each material was tested 10 times and every test was performed in standard atmospheric conditions: a temperature of 20 ± 2 °C and a room humidity of 65 ± 5%. Before testing, every sample’s diameter was measured with a caliper in order to read the results in terms of the maximum stress (MPa). The main characteristics measured were the maximum stress (MPa), elongation at break (%), and Young’s modulus (GPa).

## 3. Results and Discussion

The discussion is divided into three parts. First, the influence of spinning on the morphology and mechanical properties of the various blends is highlighted. Then, the degradation of extruded monofilaments and melt-spun filaments is discussed. Finally, the impact of the shape of the extruded and melt-spun monofilaments on their degradation is discussed.

### 3.1. PLA/PCL Blends’ Characteristics

#### 3.1.1. Morphology of the Extruded Monofilaments and Melt-Spun Filaments

**Figure 1:**
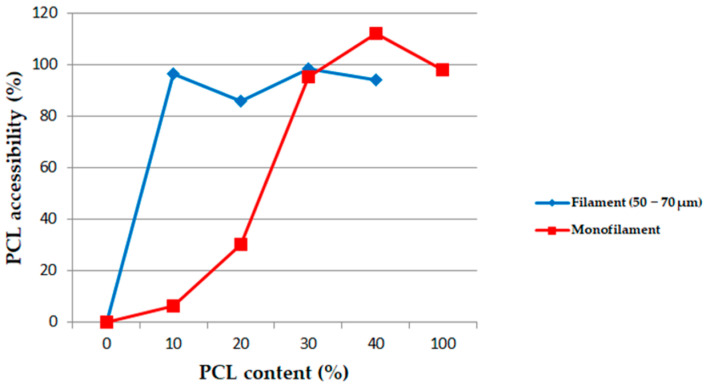
Polycaprolactone PCL accessibility (%) in poly(d,l-lactide) (PLA)/PCL extruded monofilaments and melt-spun filaments in acetic acid.

The PCL accessibility’s dependence on the PCL content in the PLA/PCL blends is illustrated in Figure 1. One curve represents the PCL accessibility in an extruded monofilament cut into small compounds (squares), while the other one shows PCL accessibility in the corresponding melt-spun multifilament (diamond).

**Figure 2:**
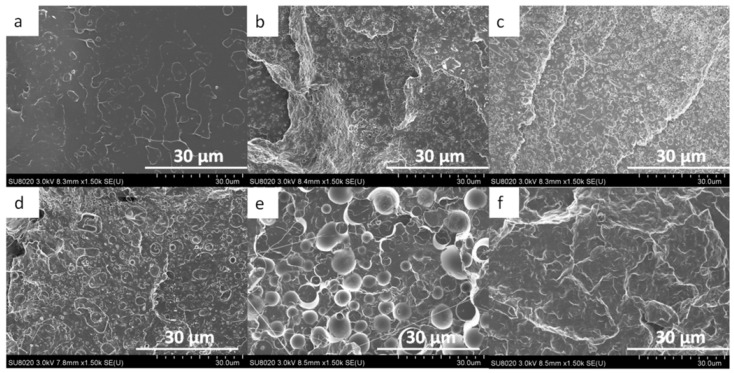
SEM images of the cross-section view of the monofilament of (**a**) neat PLA, (**b**) PLA_90_/PCL_10_, (**c**) PLA_80_/PCL_20_, (**d**) PLA_70_/PCL_30_, (**e**) PLA_60_/PCL_40_, and (**f**) neat PCL.

**Figure 3:**
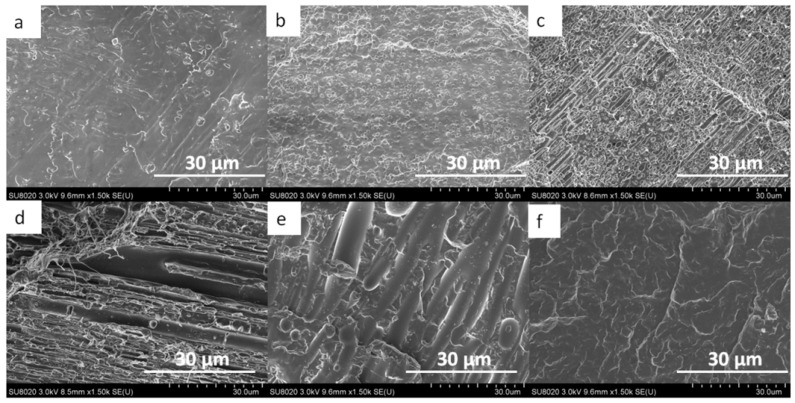
SEM images of the longitudinal view of (**a**) neat PLA, (**b**) PLA_90_/PCL_10_, (**c**) PLA_80_/PCL_20_, (**d**) PLA_70_/PCL_30_, (**e**) PLA_60_/PCL_40_, and (**f**) neat PCL.

For the extruded monofilaments containing 0 to 30 wt% of PCL in the PLA/PCL blends, the rate of extracted PCL mass was less than 100%, which means that not all the PCL was reachable with the acetic acid. In other words, some of the PCL was trapped in the PLA matrix. The SEM images also confirmed this observation (Figure 2 and Figure 3). Indeed, it can be observed in the cross-section and longitudinal views that the PCL phase was organized in small droplets, especially in the PLA_90_/PCL_10_ blend. Moreover, the morphology of PLA_80_/PCL_20_ was revealed to be different: even if the size of the particles in this blend was similar to those in PLA_90_/PCL_10_, the droplets were stretched in the longitudinal view, which was a consequence of the extrusion process. The morphology described above is usually referred to as “nodular” in the literature [22].

For the blend containing 30 wt% of PCL (PLA_70_/PCL_30_), the particles were elongated, leading to a “fibrillary” morphology (Figure 3d). Finally, for the last blend studied in this work, namely, 40 wt% of PCL (PLA_60_/PCL_40_), the phase morphology was co-continuous. These SEM images are supported by the PCL accessibility of over 100%, which means that PLA particles were trapped in the PCL matrix.

The diamond curve (Figure 1) shows the PCL accessibility in the different multifilaments that were obtained using a melt-spinning process. The results show a very high PCL accessibility in each blend containing at least 10 wt% of PCL. This indicates the impact of the drawing effect of the spinning process on the PCL accessibility, leading to elongational stress. It improved the parallelism of the macromolecules and increased the co-continuity of phases, the specific surface, and the surface-to-volume ratio. This point will be discussed in Section 3.3 of the analysis.

#### 3.1.2. Mechanical Properties

**Figure 4:**
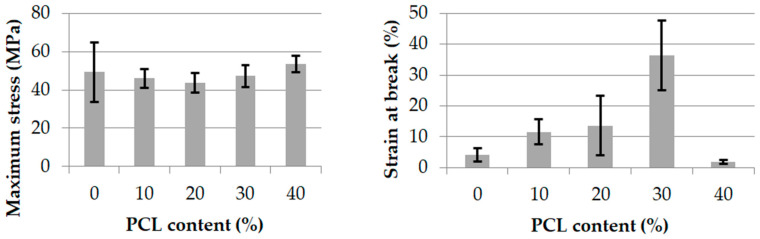
Maximum stress (MPa) and strain at break (%) of PLA/PCL extruded monofilaments.

The maximum stress and strain at break for extruded monofilaments of each blend are illustrated in Figure 4. As expected, the maximum stress did not significantly change with the increase of PCL content. However, even if the neat PLA strain at break was low, it increased with the PCL content up to 30 wt% added. In the co-continuous blend with 40 wt% PCL, this property strongly decreased. The same trend with a very low strain at break was observed by Navarro-Baena et al. [11] for neat PLA and co-continuous blends. Additionally, the highest strain at break was found for the blend containing 30 wt% PCL. The authors explain these results in terms of a poor adhesion between both polymers in a co-continuous morphology.

**Figure 5:**
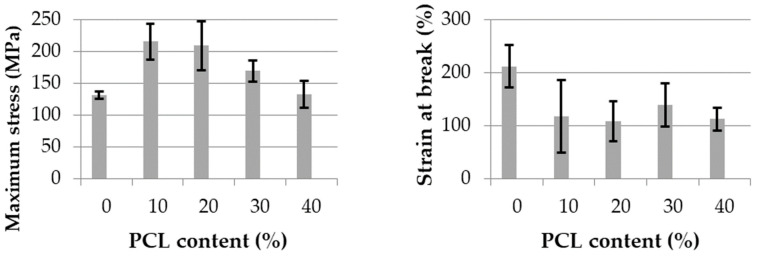
Maximum stress (MPa) and strain at break (%) of the PLA/PCL melt-spun filament extracted from the multifilament.

The same mechanical properties for the melt-spun multifilaments of the same compounds are shown in Figure 5. An overall increase in maximum stress was noticed, with a minimum of over 100 MPa for neat PLA and co-continuous PLA_60_/PCL_40_. The filaments presenting the best mechanical properties were PLA_90_/PCL_10_ and PLA_80_/PCL_20_. This last blend was found to be the most optimized composition in terms of the maximum stress according to Ostafinska et al. [23]. This substantial increase was directly linked to the increase of the macromolecules’ orientation due to the drawing out during the melt-spinning process and its impact on the macromolecules’ organization, as explained in the morphology analysis. 

The strain at break of the melt-spun filaments was also higher than that of the extruded monofilaments, especially for the neat PLA, which is a surprising result when compared to literature. Indeed, most of the time, PLA is very brittle, and when 10 wt% PCL is added, a very clear change in fracture behavior is observed. Urquijo et al. [21] demonstrated that neat PLA is brittle with a strain at break close to 0%, and when 10 wt.% or more PCL is added, the strain at break increases until 100% and more, exactly as shown in Figure 5.

### 3.2. Degradation Study

#### 3.2.1. Extruded Monofilament

**Figure 6:**
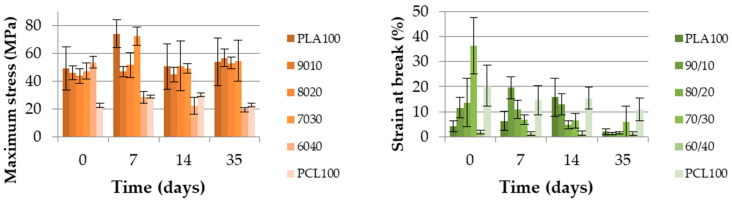
Maximum stress (MPa) and strain at break (%) of the PLA/PCL extruded monofilaments according to time.

Figure 6 displays the evolution of the maximum stress and strain at break of the extruded monofilaments over 35 days at 50 °C. It is interesting to notice that there was no decrease in the maximum stress for any blend, except for the co-continuous PLA_60_/PCL_40_ blend. Indeed, this value decreased until reaching the neat PCL value in seven days. This result indicates a poor cohesion at the interface between the PLA and PCL in a co-continuous blend. This also highlights that PLA was not playing its mechanical role anymore once degradation occurred. PCL controlled the maximum stress in this blend.

The strain at break decreased for almost every blend. As expected for neat PCL, its value remained constant. The degradation behavior of the co-continuous PLA_60_/PCL_40_ blend must be pointed out. In fact, the lack of cohesion between the PLA and PCL phases generated a very low strain at break value. This value was so low that it could no longer significantly change, even after 35 days of degradation.

The poor strain at break and the maximum stress dropping to the neat PCL values were two indications used to conclude that the interface between the PLA and PCL was poor. This finding was supported by the study of Broz et al. [24], who showed that there was little adhesion between the PLA and PCL for only the nodular morphology and the quality of the interface was higher when PCL was the matrix. Therefore, the nodular and fibrillary morphologies have to be separated from co-continuous morphologies in order to explain the degradation results. The other blends, those that were mainly composed of PLA did not present a variation of their maximum stress. However, their strain at break decreased.

**Figure 7:**
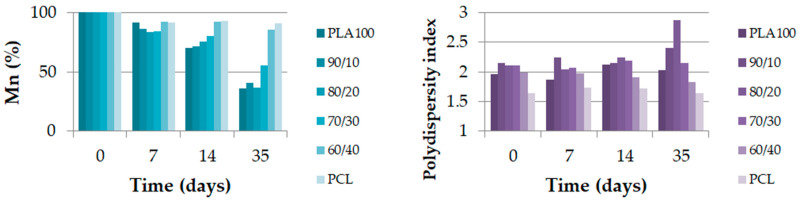
Molecular weight (Mn, %) and polydispersity index of the extruded monofilament according to time.

**Figure 8:**
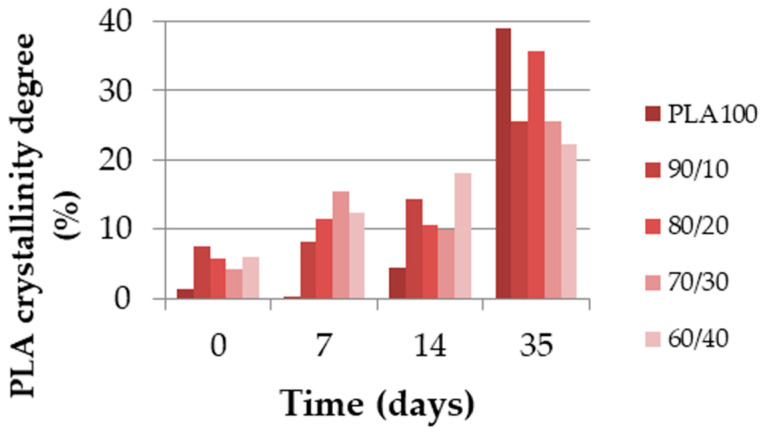
PLA crystallinity degree (%) of the extruded monofilaments according to time.

The GPC and DSC test results show how the different physical properties evolved over the degradation time. Figure 7 shows the molecular weight (Mn) as a percentage and the polydispersity index (PDI) of each blend during the degradation. The molecular weight is displayed out of 100 because the reference molecular weights were different due to the PCL amounts. Figure 8 shows the degree of crystallinity of the PLA part during the degradation. As shown in Figure 7, the molecular weight of neat PLA was seriously decreased after 35 days. The same trend was noticed for blends with 0, 10 and 20 wt% of PCL, with a remaining Mn proportion of 40% or less. Furthermore, for PLA_70_/PCL_30_, the molecular weight decreased slowly, and for PLA_60_/PCL_40_ and neat PCL, the molecular weight did not decrease to lower than 80% of the initial value. The evolution of the polydispersity index during the degradation is presented in Figure 7. It shows that neat PLA and PCL, as well as PLA_60_/PCL_40_, which presents a co-continuous morphology, were degraded in a homogeneous way and this was not the case for the nodular and fibrillar morphologies. Indeed, the polydispersity index reached 2.9 at day 35 for the PLA_80_/PCL_20_ blend. This phenomenon was explained by Sengupta et al. [25]: PCL acts as a nucleating agent that increases the crystalline ability of the PLA matrix.

Figure 8 shows very clear results about the PLA crystallinity degree. This property increased over time in every blend, especially in the case of neat PLA, which increased from almost 0 to 40%. The crystallinity degree of every other blend was under 10% and increased to more than 20% after 35 days. This was explained by Tsuji et al. [20] as an increase in the PLA chain mobility due to the decrease in molecular weight during the hydrolysis of ester groups. As a consequence, the crystallinity degree of PLA increases.

#### 3.2.2. Filaments Extracted from the Multifilaments

**Figure 9:**
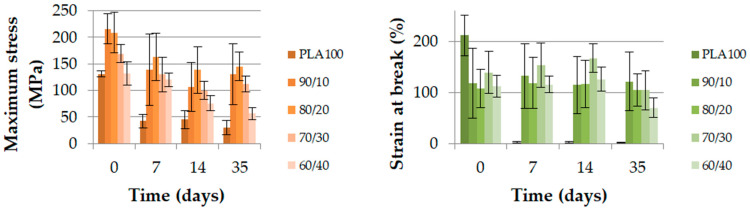
Maximum stress (MPa) and strain at failure (%) of the PLA/PCL melt-spun filament extracted from the multifilaments according to time.

The maximum stress and strain at break for the filaments extracted from every multifilament of each blend are displayed in Figure 9. The main observation was the rapid decrease in maximum stress for each blend during the first 14 days, and then, a plateau seemed to occur from 35 days onward. For the maximum stress analysis, blends could be separated into three categories: the neat PLA; 10 wt%, 20 wt%, and 30 wt% PCL content blends; 40 wt% PCL content blend. The neat PLA displayed a very fast decrease during the first week and then stabilization of the maximum stress, while the 10 wt%, 20 wt%, and 30 wt% PCL content blends had a slower decrease than the neat PLA, but also plateaued after 2 weeks. Finally, the 40 wt% PCL content blend had a regular decrease, with an ultimate value close to the neat PLA maximum stress. The strain at break had a different evolution compared to the extruded monofilament for most of the blends. In the case of neat PLA, the filament with the highest strain at break lost this property over the first week. The 10 wt%, 20 wt%, and 30 wt% PCL content blends remained constant over time. Finally, the 40 wt% PCL content blend lost the same amount of strain at break as the extruded monofilament.

**Figure 10:**
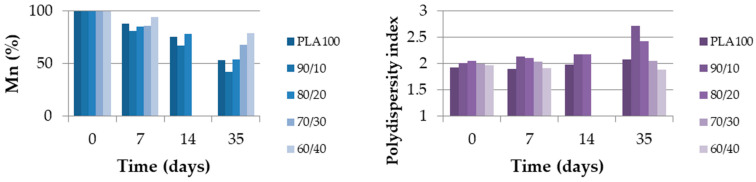
Molecular weight (Mn, %) and polydispersity index of the melt-spun filaments extracted from the multifilaments according to time.

Even though the molecular weights of the 10 wt%, 20 wt%, and 30 wt% PCL blends strongly decreased (Figure 10), the maximum stress over time of these blends was the best. After one month, the polydispersity index of the 10 wt% and 20 wt% PCL blends increased, which suggests that the hydrolytic degradation was heterogeneous. This phenomenon could explain the maintenance of the mechanical properties over time for these two blends. Despite the evolution of the neat PLA crystallization over time, its polydispersity index remained constant. This finding was already explained in the literature in terms of the bulk degradation of PLA [26]. 

**Figure 11:**
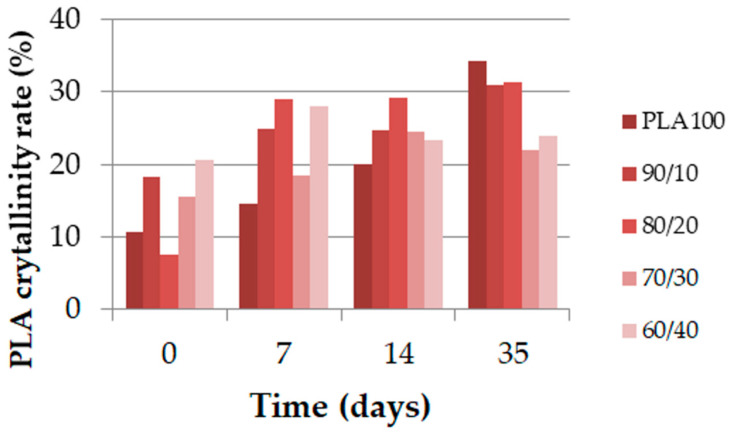
PLA crystallinity degree (%) of the melt-spun filaments extracted from the multifilaments according to time.

An other main observation to be made is that in the filament form, the molecular weight of the PLA/PCL blends decreased slower than the extruded monofilament form, except for PLA_60_/PCL_40_, where a similar trend for both material forms was observed. One explanation is the initial difference in crystallinity degree between the extruded monofilament and melt-spun filaments: Figure 8 shows that the value did not exceed 10% for each blend, whereas it was between 10% and 20% for the melt-spun filaments (Figure 11), which had a faster increase in the extracted filaments in the second week.

It was also noticed that the three blends with the best mechanical performances (PLA_90_/PCL_10_, PLA_80_/PCL_20_, and PLA_70_/PCL_30_) after one month of degradation were the blends organized as nodular and fibrillar morphologies.

### 3.3. Discussion on the Influence of the Two Types of Shapes: Extruded Monofilament and Melt-Spun Filament

In the literature, many discussions can be found about the influence of the size of the sample on its degradation behavior [27]. The thickness of the biodegradable polymer samples usually drives the degradation. However, as explained in the introduction, some authors have been working on a model that predicts whether a polymer will undergo surface or bulk degradation [16], depending on a critical thickness, L_critical_. If the material thickness is under this critical value, it will generate bulk degradation, and if the thickness is over this value, the material will undergo surface degradation. Therefore, the surface-to-volume ratio is an important parameter that drives degradation [12]. This thickness value was found to be 7.4 cm for PLA and 1.3 cm for PCL in this model, which was much higher than the extruded monofilament cross-section thickness (1 mm) and obviously higher than the filaments’ cross-section thickness (50–70 µm). According to this model, the extruded monofilaments and melt-spun filaments that were studied were supposed to undergo bulk degradation. This was verified using the different polydispersity index (Figure 7 and Figure 10), which stayed constant over time. For a non-constant polydispersity value (10 and 20 wt% PCL content), this was related to morphology and compatibility between the PLA and PCL, as explained earlier in the analysis.

Although each blend of both forms underwent bulk degradation, some important differences were pointed out based on the various characterizations performed over time. For instance, the mechanical properties evolved very differently over time depending on the polymer sample form. The monofilaments had a suitable maximum stress, while it decreased for filaments. However, a significant loss of strain at break was observed, while it was slower for filaments. Another difference that was pointed out was the evolution of the molecular weight during degradation: it was faster for the extruded monofilaments than the filaments from 0 to 30 wt% PCL content in the blend. These differences meant that the model considered was therefore not sufficient to understand the degradation of these two different polymer sample forms.

One first element that is consistent with the model of Von Burkersroda [16] is the relative importance of the surface-to-volume ratio under L_critical_ in comparison with the production process (extrusion and yarn spinning) and the autocatalysis of PLA degradation. As shown before, the yarn melt spinning process had an impact on the morphology, which influenced the initial PLA crystallinity and their mechanical properties. The autocatalytic aspect of the PLA hydrolysis is described in the literature, especially by Siparsky et al. [18], who proposed a model for PLA and PCL degradation behaviors. They pointed out the autocatalytic degradation of PLA and its first-order degradation kinetics. Some studies regarding the size-dependence of PLA on its degradation were carried out. One of the oldest ones, performed by Grizzi et al. [15], already proved that this physical principle of autocatalysis was verifiable and reliable for large plates. These large plates could degrade faster than submillimetric films and particles.

These studies provided a better understanding of how extruded monofilaments (~1 mm) could degrade faster than filaments (~50–70 μm). The surface-to-volume ratio might not have an influence on water diffusion in a polymer shaped under L_critical_, but it is important to estimate the impact of the autocatalysis of PLA. In this study, this ratio was 17 times higher for a melt-spun filament than an extruded monofilament.

**Figure 12:**
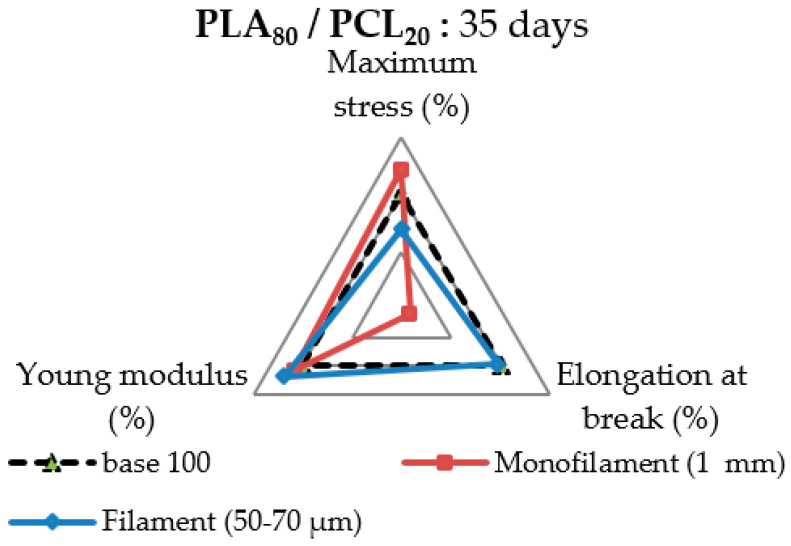
Mechanical properties of the PLA_80_/PCL_20_ blend after 35 days of degradation in comparison with an extruded monofilament and a melt-spun filament as a percentage.

As a result, textile melt-spun filaments could create sufficient interest when it comes to designing a 3D-scaffold. It is possible to enhance the scaffold lifespan through melt spinning because of its impact on morphology, and thus, on the crystallinity and mechanical properties. The filaments are thin, which will limit the autocatalysis of PLA compared to a 3D-printed system, for example. Figure 12 illustrates the advantage of using a textile multifilament compared to the extruded monofilament shape over time for possible bioresorbable applications. The residual mechanical properties of a PLA_80_/PCL_20_ filament and extruded monofilament after 35 days of degradation are displayed as a percentage of the reference properties of each shape. The filament degradation was more balanced than the extruded monofilament degradation, with a slight decrease in Young’s modulus, maximum stress, and elongation at break. The finding might be interesting for better design and control of the properties of a 3D-scaffold after degradation. However, the extruded monofilament quickly lost elongation at break down to less than 10% of the initial value, despite maintaining its Young’s modulus and maximum stress.

## 4. Conclusions

In this study, immiscible PLA/PCL blends were prepared via extrusion and melt-spinning processes in order to study the influence of the morphology on the blends’ physico-chemical and mechanical properties. Then, in vitro degradation was performed to understand the degradation behavior differences between the extruded monofilaments and multifilaments. The results revealed the importance of morphology on mechanical properties, and the consequent increase in mechanical properties when using a melt-spinning process. Moreover, the differences in the evolution of the mechanical properties during degradation were highlighted, such as the decrease in the strain at break for the extruded monofilament and a more balanced degradation for the filaments. The importance of the process and shape of the polymer blend for the degradation was also examined. This allowed us to understand the differences in crystallinity degree, which was higher for multifilaments than extruded monofilaments. Therefore, the molecular weight during degradation decreased slower for multifilaments. Possible applications of melt-spun filaments for the design of 3D-scaffold applications were highlighted in this work.

## Data Availability

Data available on request due to restriction eg privacy or ethical.
